# Supplementary material for: Attracting Cavities 3.0: faster and more versatile molecular docking for the SwissDock webserver
Source: Bioinformatics. 2026 Aug 21;42(Suppl 2):btag466. doi: 10.1093/bioinformatics/btag466 (PMC13501311; doi:10.1093/bioinformatics/btag466)
Supplement: Supplementary file 1 [file btag466_supplementary_data.pdf]

# – Supplementary Material –

Attracting Cavities 3.0: Faster and More Versatile Molecular Docking for the SwissDock Webserver

Ute F. Röhrig[1,*], **Marine Mathieu-Bugnon**[1], **and Vincent Zoete**[1,2,*]

[1]SIB Swiss Institute of Bioinformatics, Molecular Modeling Group, CH-1015 Lausanne, Switzerland

[2]Department of Oncology UNIL, Lausanne University, Ludwig Institute for Cancer Research Lausanne Branch, CH-1066 Epalinges

[*]ute.roehrig@sib.swiss, vincent.zoete@unil.ch

List of Tables

S1 Stereochemistry Analysis . . . 2

List of Figures

S1 AC 2.0 Sampling Options . . . 3
S2 Comparison of AC 2.0 and AC 3.0 . . . 4
S3 Evaluation of Old and New AC Sampling Options . . . 5
S4 Evaluation of Equivalent Sampling Options . . . 6
S5 Evaluation of Active Site Focus . . . 7
S6 Covalent Docking Results . . . 8
S7 Comparison of MMFF and MATCH Ligand Parameters . . . 9
S8 Evaluation of Cavity Prioritization Value . . . 10
S9 Comparison of AC and Vina on Astex Set . . . 11
S10 Comparison of AC and Vina on RNP and RNP-F Sets . . . 12
S11 Comparison of AC 3.0 and AutoDock Vina . . . 13

Supporting Tables

Set	Stereo [%]	Chiral carbon [%]	Double Bond [%]	Top Wrong [%]	Max Wrong [%]
**Astex**	65	56	14	0	13 (1hww)
**PDBbind**	59	55	9	1	27 (3g0w)
**RNP**	62	59	6	3	87 (7rbw)
**CSKDE56**	63	55	18	9	85 (4wsk)

Table S1. Analysis of stereochemistry across data sets. Stereo: ligands presenting at least one stereo element. Chiral carbon: ligands presenting at least one chiral carbon atom. Double bond: ligands presenting at least one stereogenic double bond. The last two columns relate to AC docking results with standard parameters (90° rotation, 4 RIC, 8 PP, 4 initial conformers). Top wrong: top poses with wrong stereochemistry. Max wrong: PDB ID and percentage of poses with wrong stereochemistry of complex with maximal percentage of poses with wrong stereochemistry. All values are given as percentages.

Supporting Figures

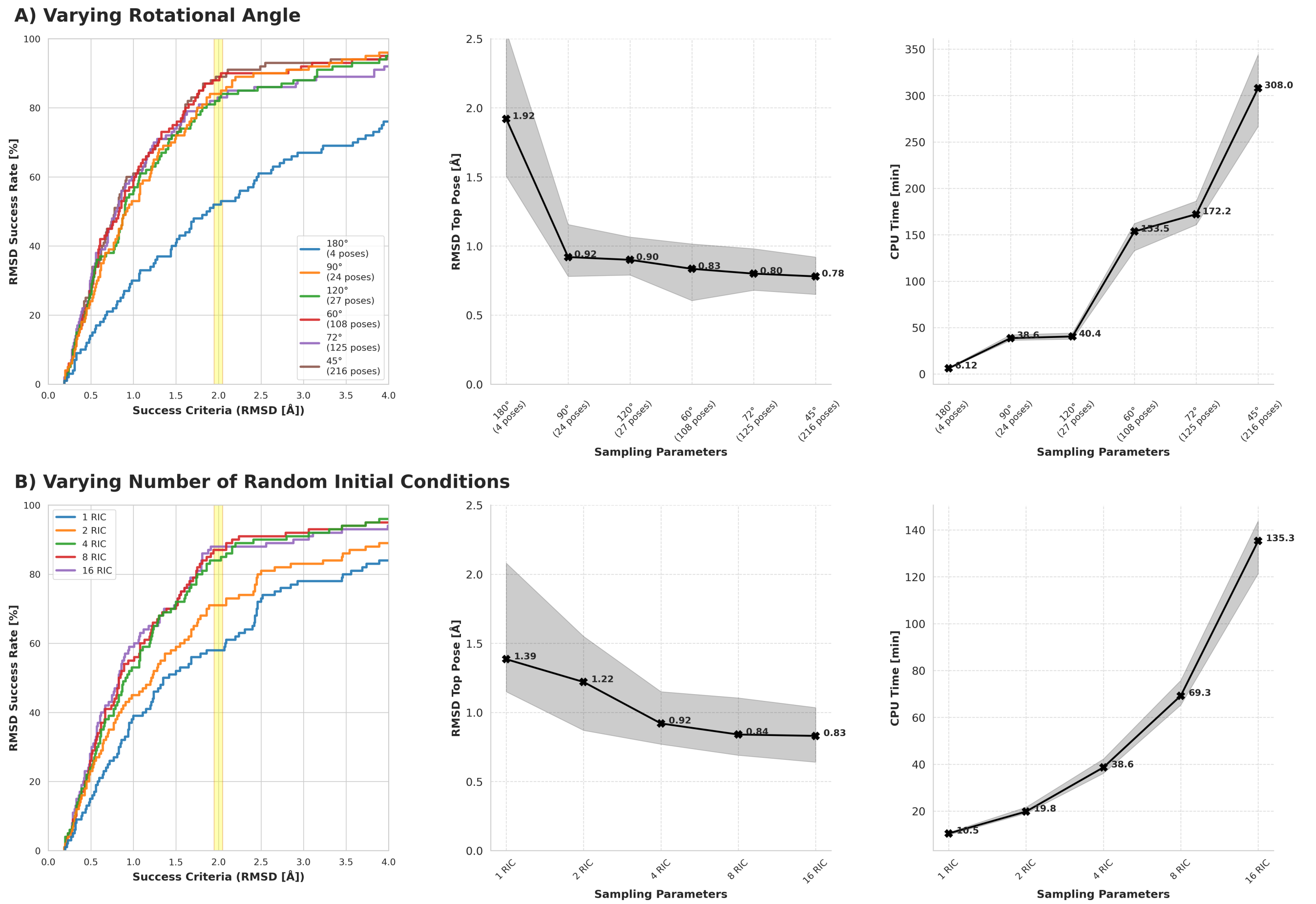

Figure S1. AC docking results on PDBbind-Sampl set of 100 complexes as a function of sampling parameters implemented in AC 2.0. A) Success rates, top pose RMSD, and CPU time as a function of varying initial rotational angle of the ligand. B) Success rates, top pose RMSD, and CPU time as a function of varying number of random initial conditions (RIC, 1–16). Shaded regions correspond to the 95% confidence interval calculated from 1,000 bootstrap samples.

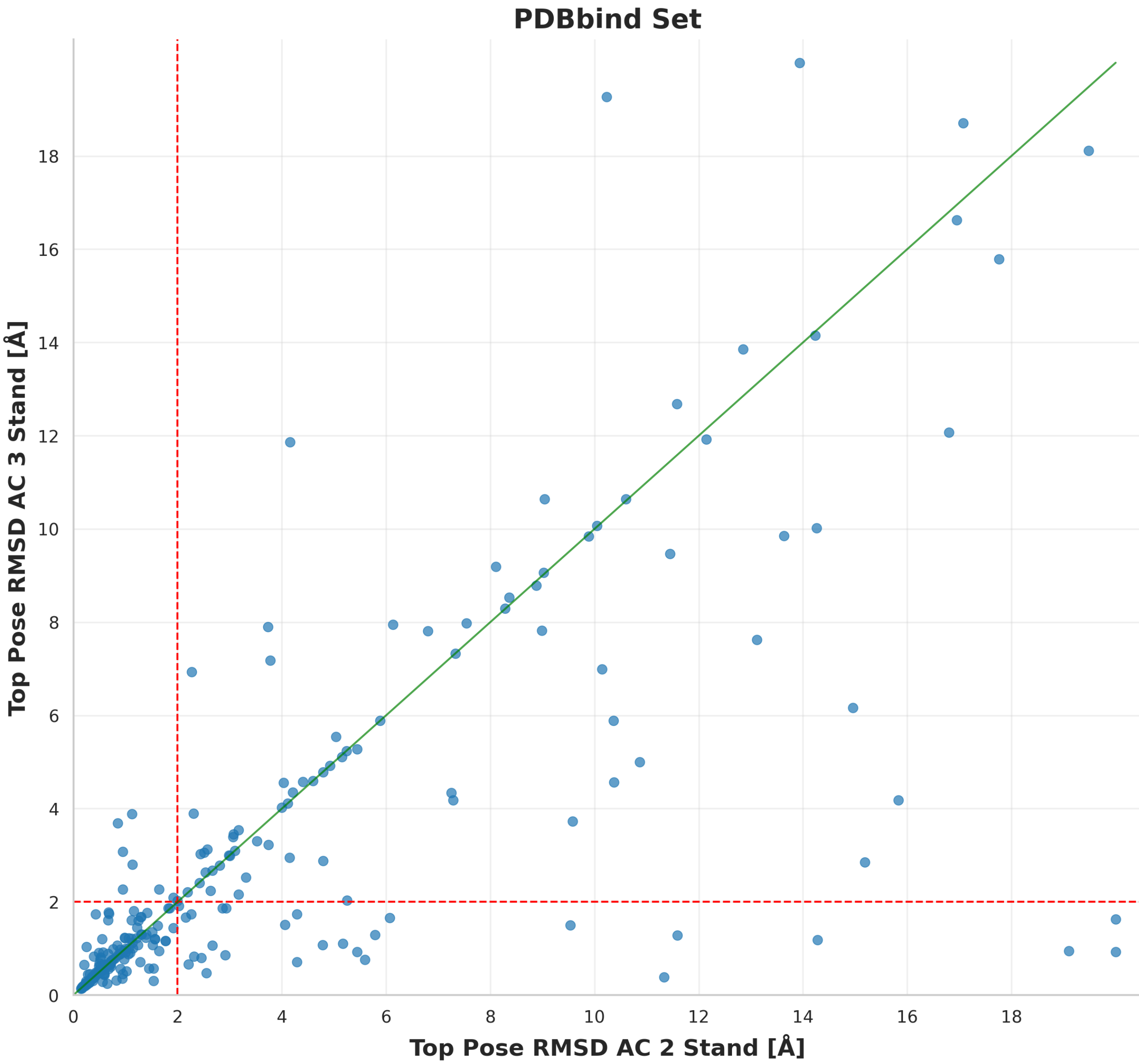

Figure S2. Comparison of top pose RMSD values of AC 2.0 and AC 3.0 with respective standard parameters on the 285 complexes of the PDBbind Set.[1] The cutoff value of 2 Å is marked by a red line.

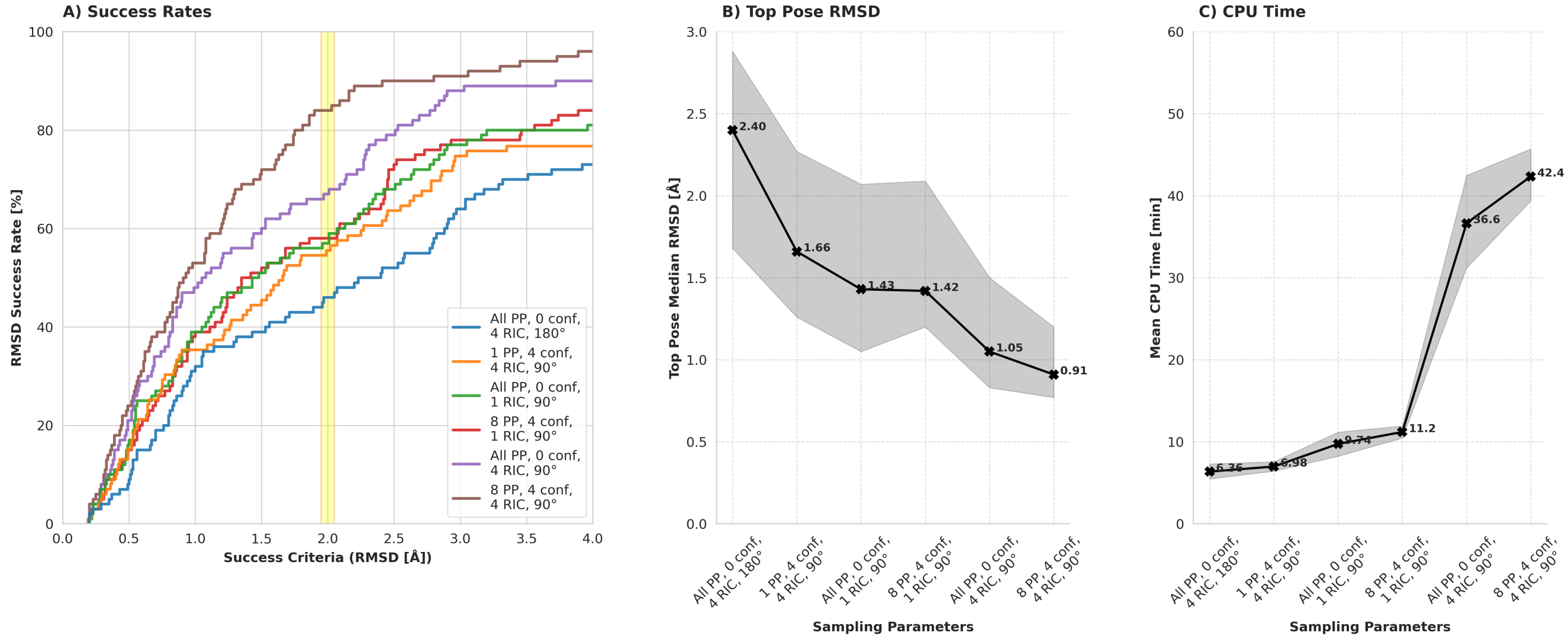

Figure S3. AC docking results on PDBbind-Sampl set of 100 complexes with only old and a combination of old and new sampling options. Dockings with similar mean CPU timings are shown for the two settings. In general, at similar CPU timings better sampling is obtained by integrating the new options.

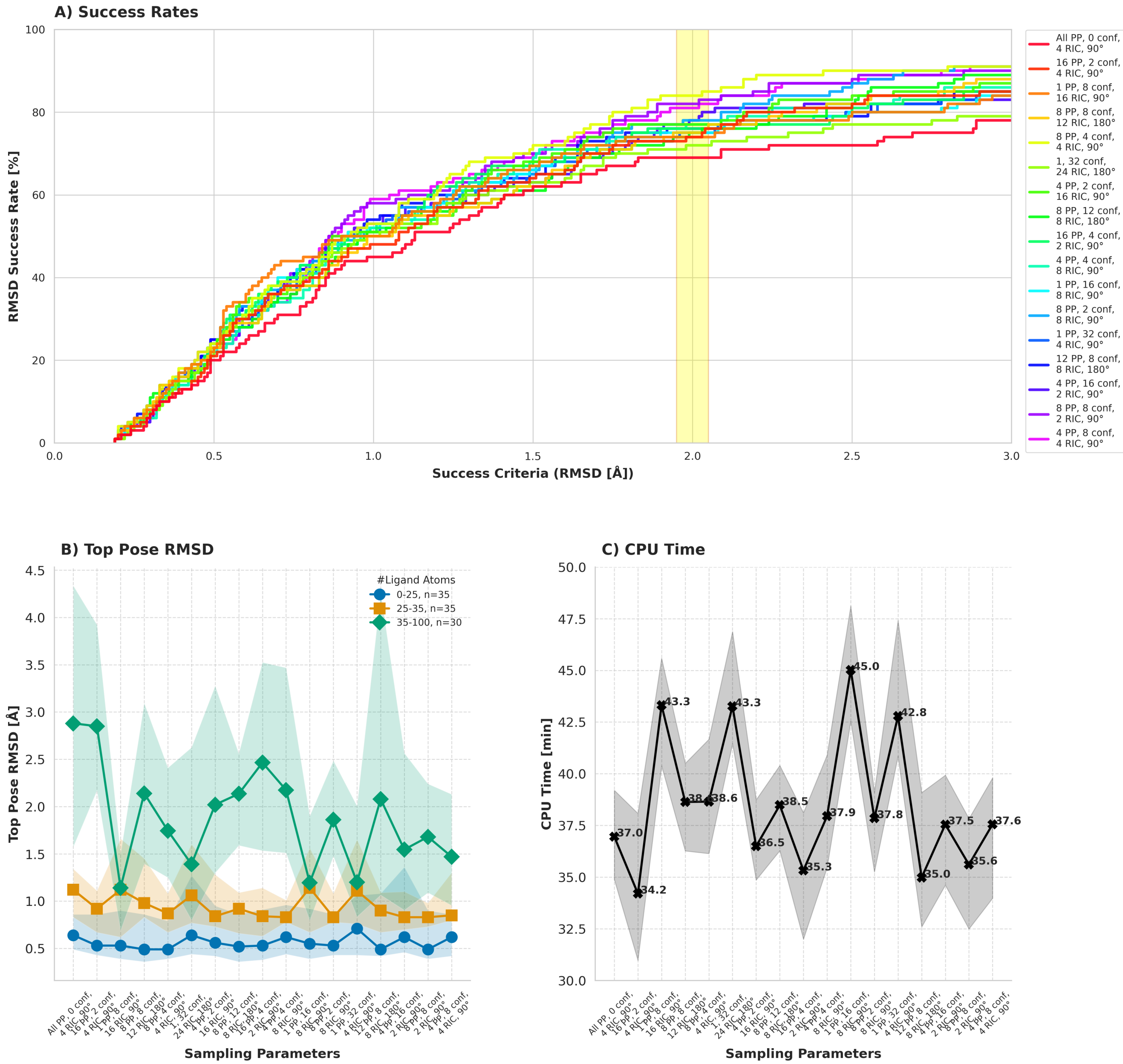

Figure S4. AC docking results on PDBbind-Sampl set of 100 complexes with options theoretically yielding 3072 starting poses. A) Success rates at different RMSD cutoffs. B) Top pose RMSD, displayed by ligand size. C) CPU timings. The biggest differences in sampling were observed for large ligands with more than 35 heavy atoms.

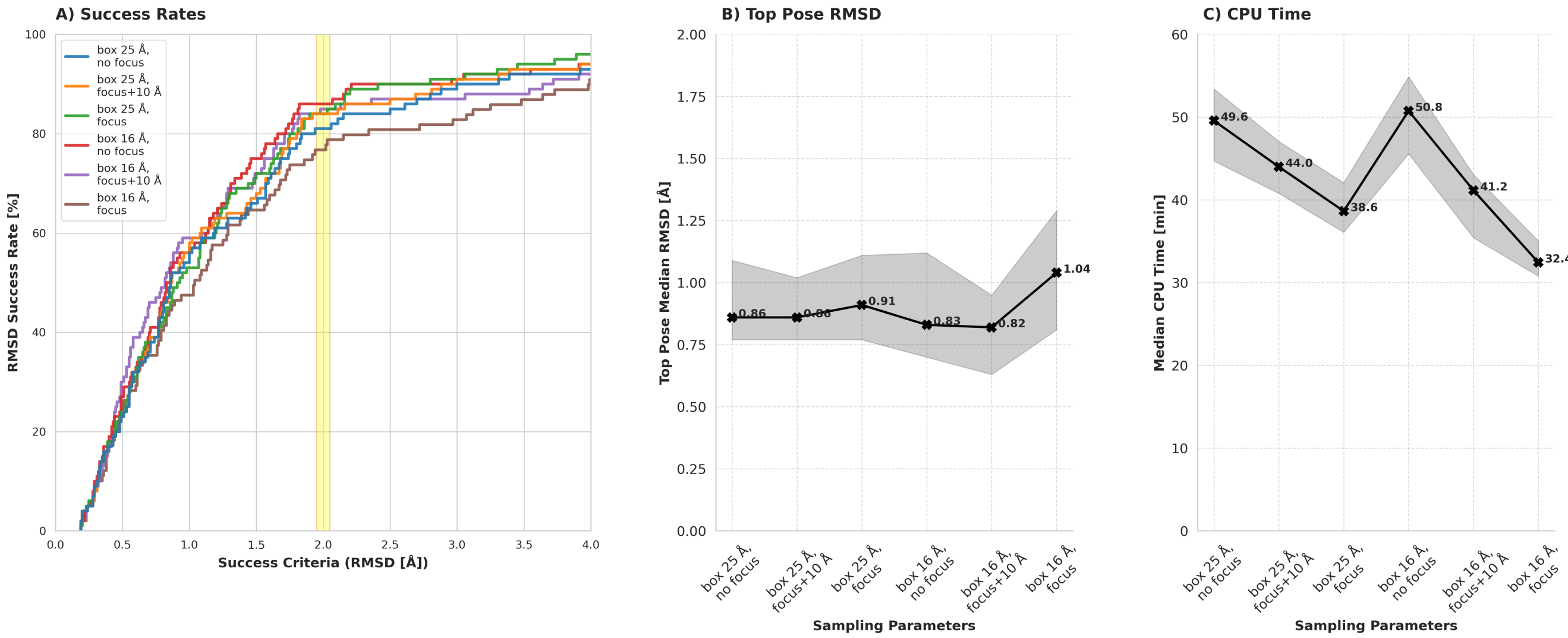

Figure S5. AC docking results on PDBbind-Sampl set of 100 complexes with and without active-site focus. Two different search space sizes, $(25\ \text{Å})^3$ and $(16\ \text{Å})^3$ were used. For each size, 3 calculations were carried out, one without focus, one with focus on the search space + 10 Å extension, and one with focus on the box without extension. A focus on the search space reduced CPU times by almost 30% without extension and about half that amount with extension. For the larger box, the success rate at 2.0 Å RMSD and top pose RMSD values remained relatively stable, while for the smaller box, a drop in successful docking predictions can be observed.

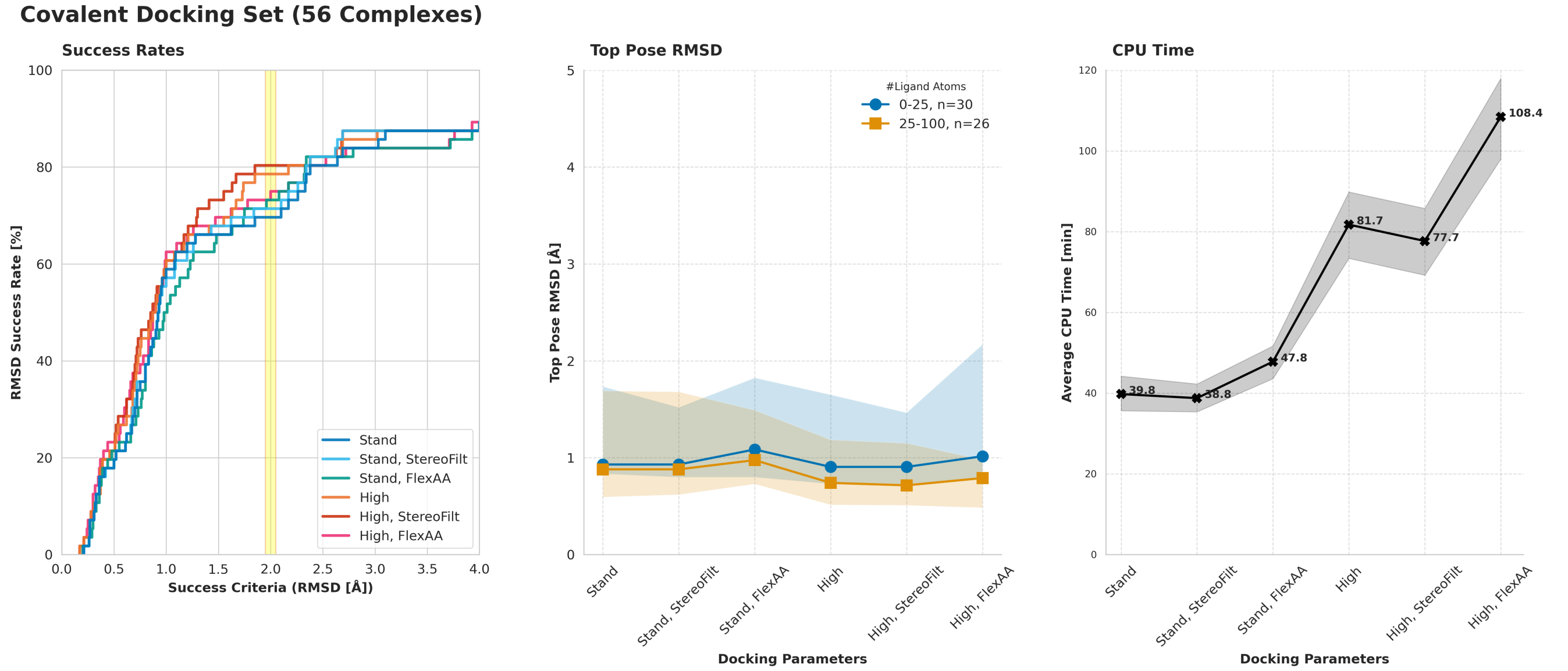

Figure S6. AC covalent docking results with standard and optimized sampling parameters. Additionally, results are shown for docking when filtering our poses with wrong stereochemistry (“StereoFilt”) and when leaving the reactive protein side chain flexible (“FlexAA”). Stereochemistry filtering does not show a significant impact on the results, while a flexible side chain leads to slightly worse results. However, this option may be very beneficial in cross-docking settings.

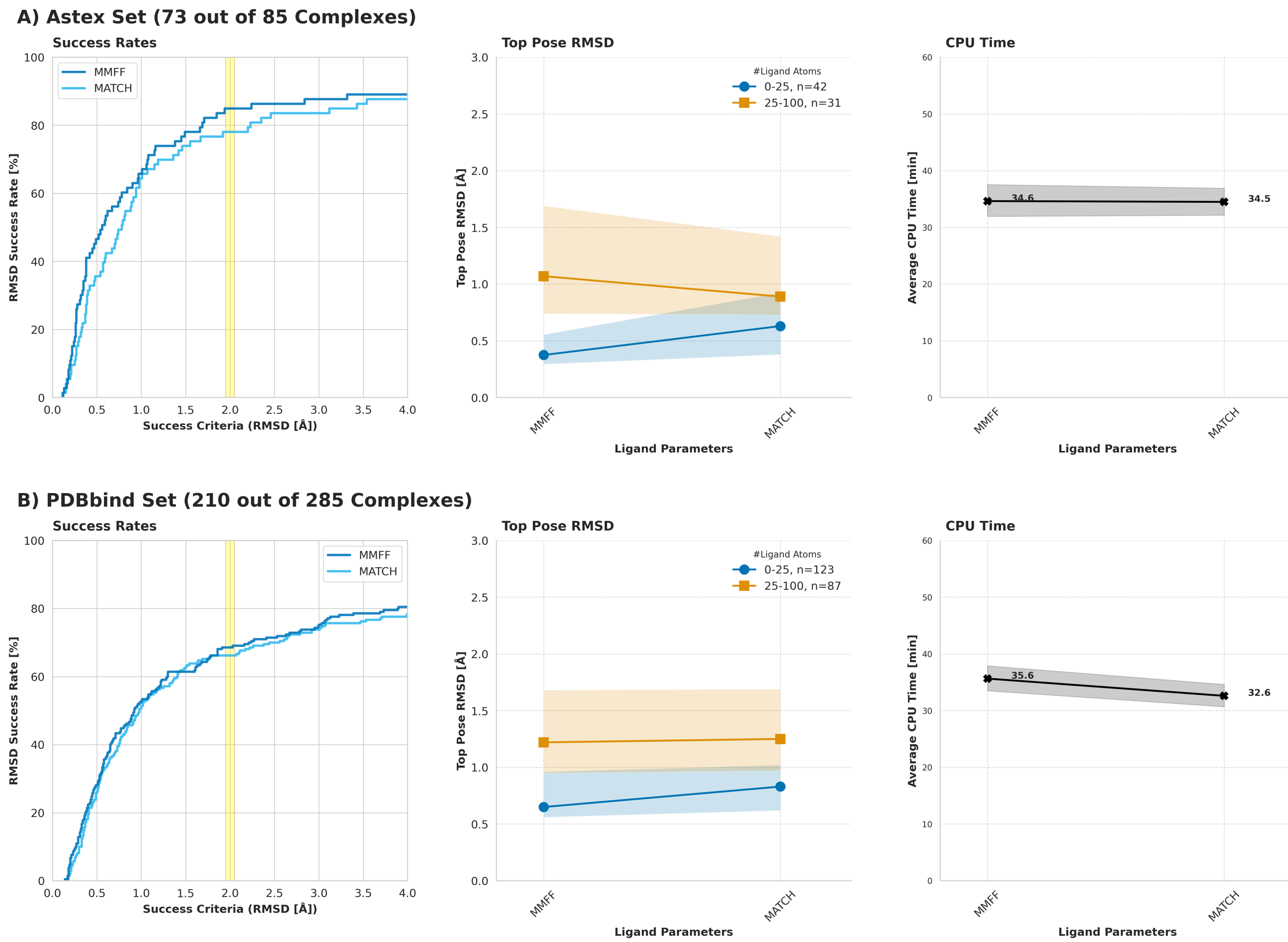

Figure S7. Comparison of AC docking results with standard sampling parameters (90° rotation, 4 RIC, 8 PP, 4 initial conformers) and with ligand parameters generated by MMFF-like[2] and by MATCH[3] approaches. A) Results on subset of Astex set, for which MATCH parameters were obtained. B) Results on subset of PDBbind set, for which MATCH parameters were obtained.

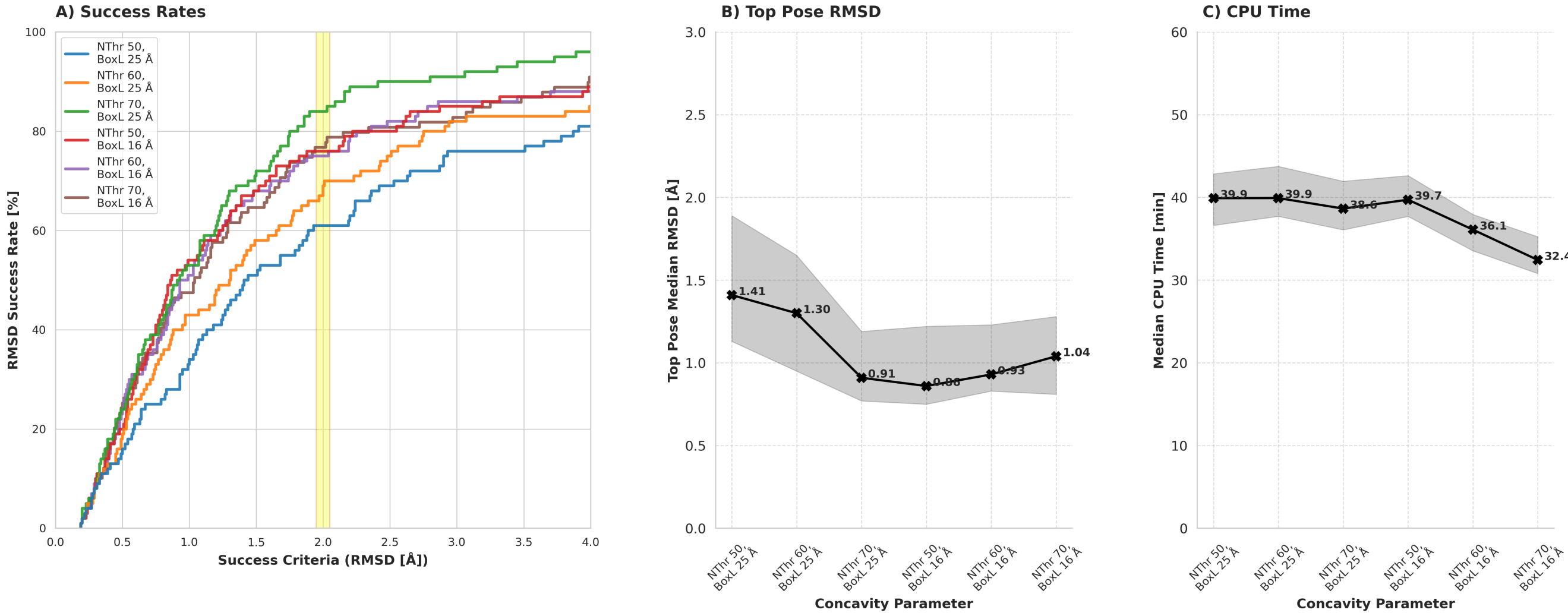

Figure S8. AC docking results on PDBbind-Sampl set of 100 complexes with different cavity prioritization values (50, 60, 70). The results demonstrate that the optimal value depends on the search box size.

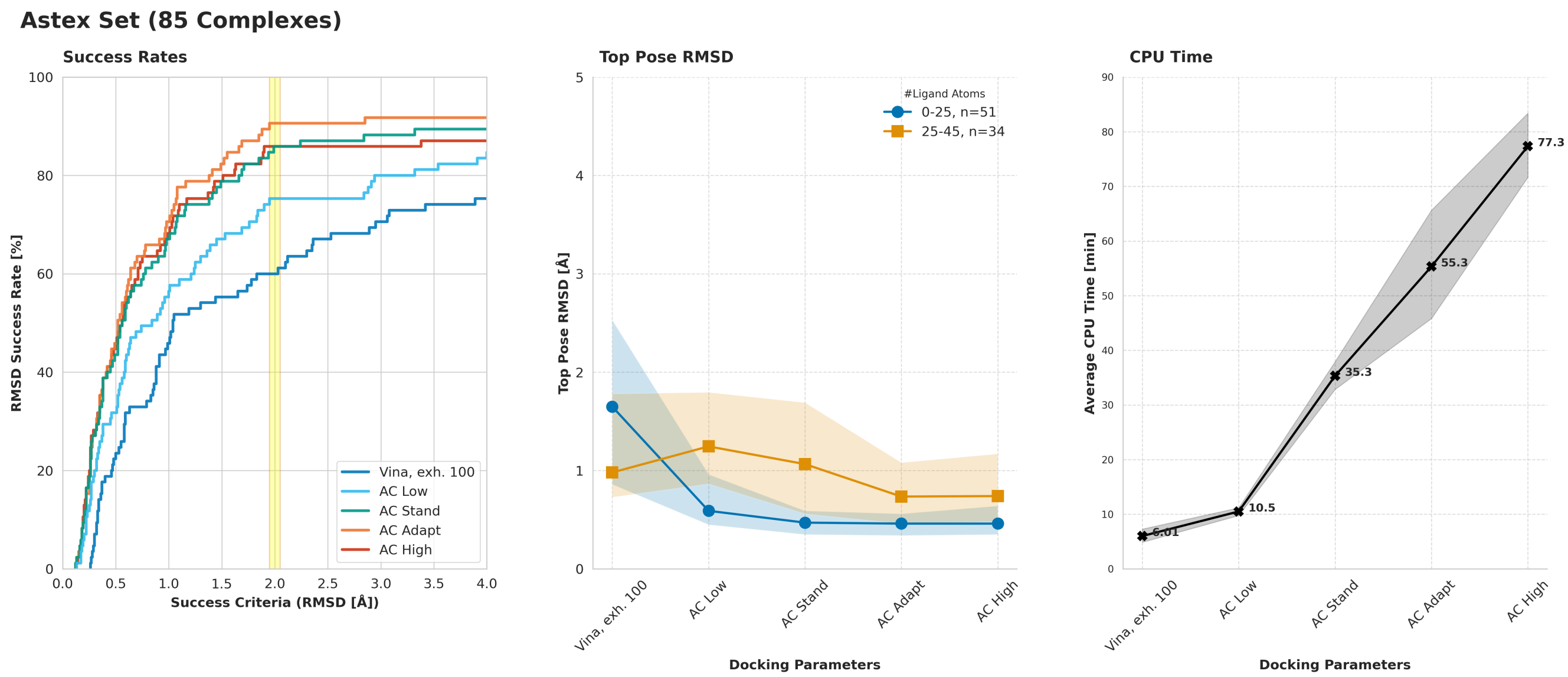

Figure S9. Comparison of docking results of AC 3.0 with AutoDock Vina on the Astex Diverse set.[4] A) Success rates. B) Top pose RMSD, displayed by number of ligand atoms. C) Average CPU times.

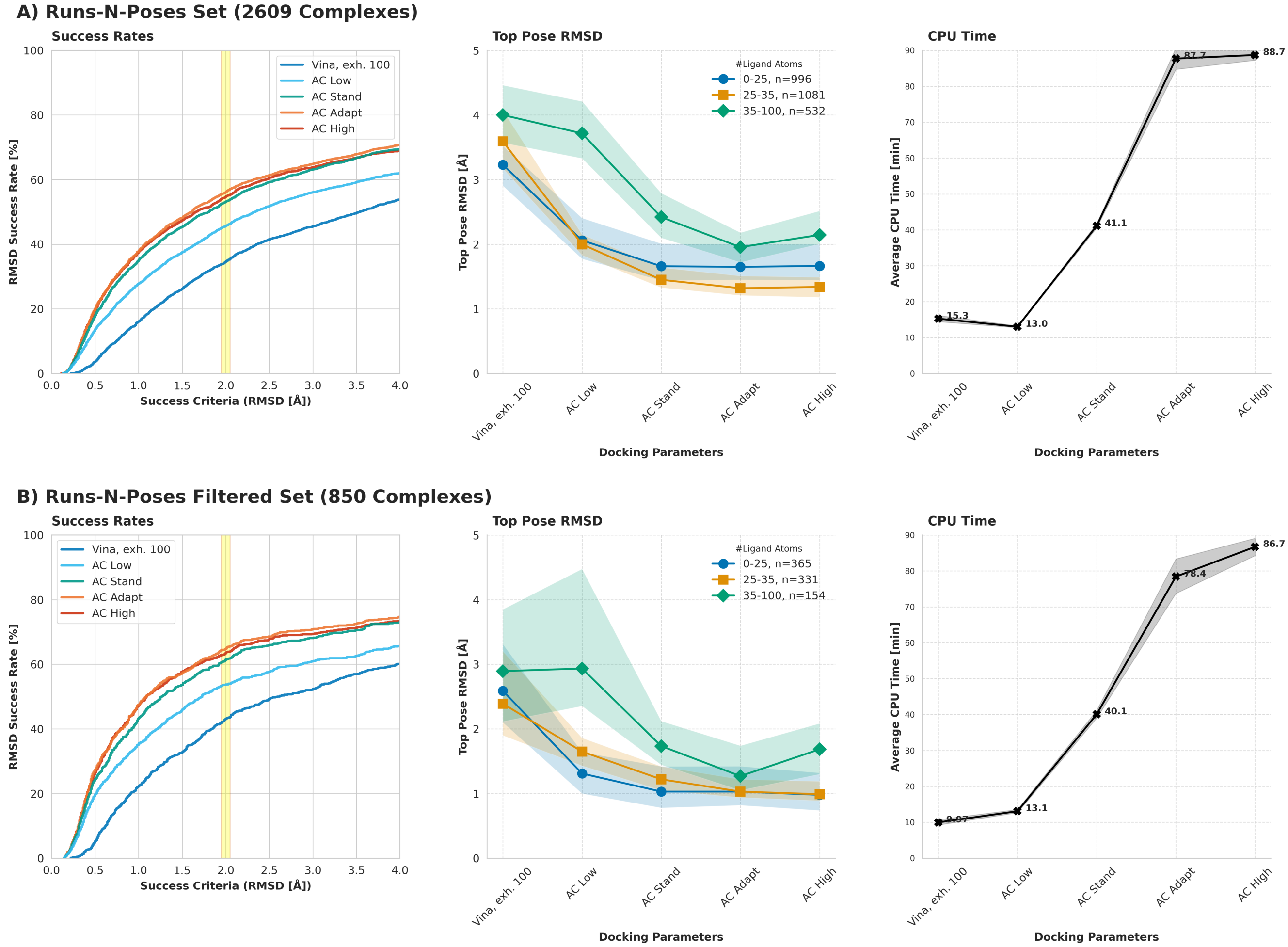

Figure S10. Comparison of docking results of AC 3.0 with AutoDock Vina A) on the RNP set[5] and B) on its version filtered for quality and diversity (RNP-F)[6].

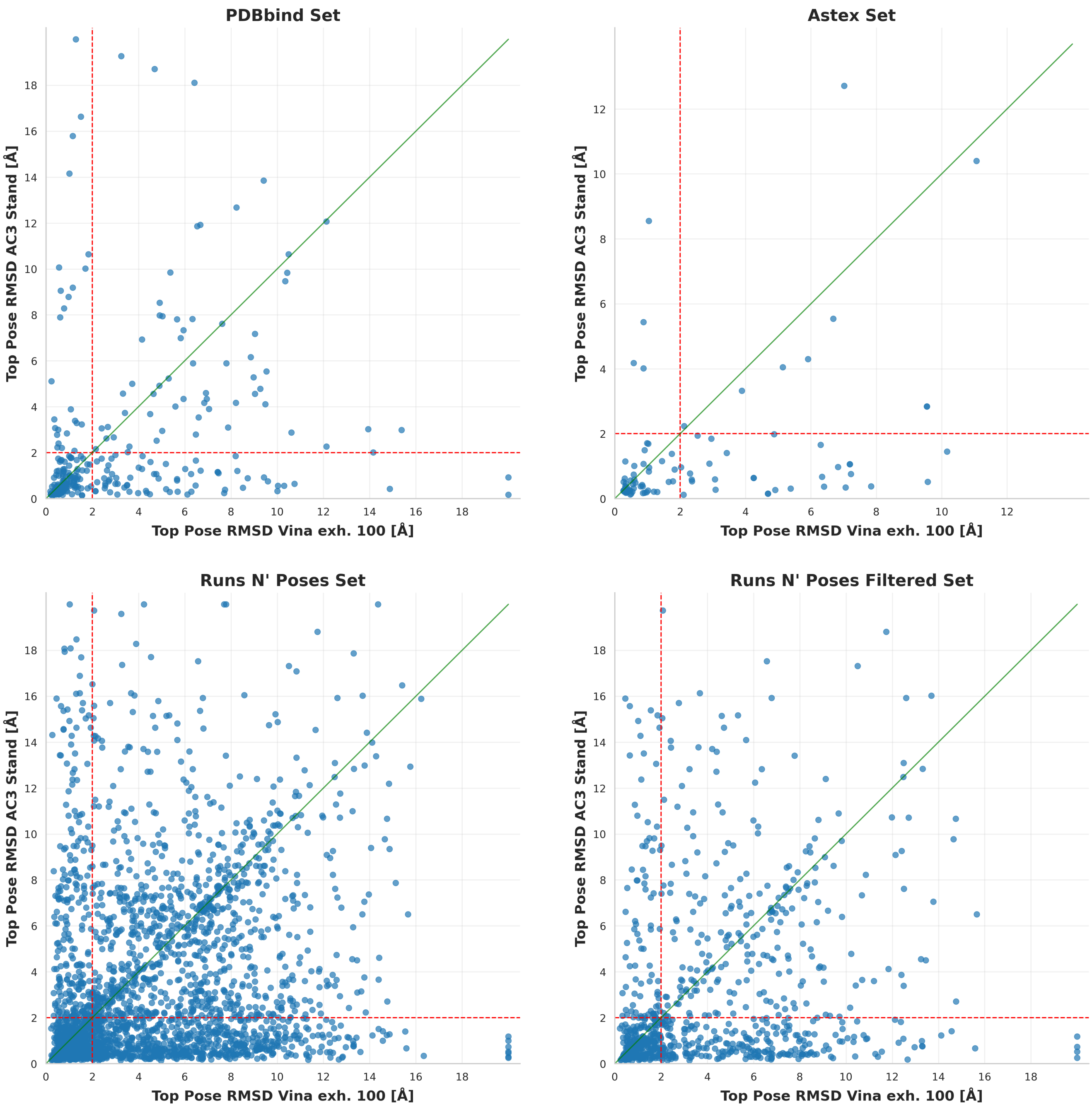

Figure S11. Comparison of top pose RMSD values of AC 3.0 with standard parameters and AutoDock Vina with exhaustivity 100 on different test sets. The cutoff value of 2 Å is marked by a red line.

References

1. Su, M. *et al.* Comparative Assessment of Scoring Functions: The CASF-2016 Update. *J. Chem. Inf. Model.* **59**, 895–913, DOI: https://doi.org/10.1021/acs.jcim.8b00545 (2019).

2. Halgren, T. A. Merck molecular force field. I. Basis, form, scope, parameterization, and performance of MMFF94. *J. Comput. Chem.* **17**, 490–519, DOI: https://doi.org/10.1002/(SICI)1096-987X(199604)17:5/6<490::AID-JCC1>3.0.CO;2-P (1996).

3. Yesselman, J. D., Price, D. J., Knight, J. L. & Brooks 3rd, C. L. MATCH: an atom- typing toolset for molecular mechanics force fields. *J Comput. Chem* **33**, 189–202, DOI: https://doi.org/10.1002/jcc.21963 (2012).

4. Hartshorn, M. J. *et al.* Diverse, high-quality test set for the validation of protein-ligand docking performance. *J. Med. Chem.* **50**, 726–741, DOI: https://doi.org/10.1021/jm061277y (2007).

5. Škrinjar, P. *et al.* Evaluating generalization in protein–ligand cofolding methods. *Nat. Struct. Mol. Biol.* **33**, 782–794, DOI: https://doi.org/10.1038/s41594-026-01797-5 (2026).

6. Röhrig, U. F., Mathieu-Bugnon, M. & Zoete, V. Comparative assessment of the utility of co-folding and docking for small-molecule drug design. *bioRxiv* 2025.12.09.693161, DOI: https://doi.org/10.64898/2025.12.09.693161 (2025).
